# BMC Anesthesiology Reviewer Acknowledgement, 2012

**DOI:** 10.1186/1471-2253-13-3

**Published:** 2013-04-11

**Authors:** Thomas A Rowles

**Affiliations:** 1 BioMed Central, Floor 6, 236 Gray's Inn Road, WC1X 8HB, London, United Kingdom

## Abstract

**Contributing reviewers:**

The editors of BMC Anesthesiology would like to thank all our reviewers who have contributed to the journal in Volume 12 (2012).

## 

Fernando Abelha

Portugal

Feray Akgül Erdil

Turkey

Roberto Arcioni

Italy

Angela Bader

USA

Alberto Barbieri

Italy

Georgia Beasley

USA

Dan Benhamou

France

Horst Bickel

Germany

Tom Birkenhäger

Netherlands

Simon Body

USA

Ben Boedeker

USA

Francis Bonnet

France

Judith Bosmans

Netherlands

Dylan Bould

Canada

Peter Bragge

Australia

Stefan Breitenstein

Switzerland

Serge Brimioulle

Belgium

Peter Brindley

Canada

Ethan Bryson

USA

Mehmet Fatih Can

Turkey

Somrat Charuluxananan

Thailand

Christian Fynbo Christiansen

Denmark

Eric Chudler

USA

Francois Clergue

Switzerland

Mark Coburn

Germany

Richard Cooper

Canada

J. Randall Curtis

USA

Giorgio Danelli

Italy

Alberto De Armendi

USA

Daniel De Backer

Belgium

Frederik De Buck

Belgium

Germano De Cosmo

Italy

Phillip Dellinger

USA

Paul D'Orazio

USA

Volker Dörges

Germany

Martin Dworschak

Austria

Sam Eldabe

United Kingdom

Thomas Engelhardt

United Kingdom

Lorella Fanti

Italy

Emanuele Ferrero

Italy

John Fiadjoe

USA

Paul Firth

USA

Dominique Fletcher

France

Elisabeth Gaertner

France

Luis Gaitini

Israel

Ronald George

Canada

Timothy Girard

USA

Allan Gottschalk

USA

Bruce Green

Australia

Burak Guclu

Turkey

Freedom Nkhululeko Gumedze

South Africa

Ashraf Habib

USA

Robert G Hahn

Sweden

Kaoru Hara

Japan

James Heavner

USA

David Herd

Australia

Jochen Hinkelbein

Germany

Luc Hondeghem

Belgium

Yu-Hsi Hsieh

Taiwan

Luiz Eduardo Imbelloni

Brazil

Fabian Jaimes

Colombia

Jerzy Jankun

USA

Bernd Jilma

Austria

Ken Johnson

USA

Hiroyuki Kinoshita

Japan

Naohiro Kurotaki

Japan

Giovanni Landoni

Italy

Tae Hoon Lee

South Korea

Hendrikus Lemmens

USA

Alyn Lewis

United Kingdom

Vladimir Lomivorotov

Russian Federation

Rebekah Lucas

USA

Marco Luchetti

Italy

Zhengliang Ma

China

Flavia Machado

Brazil

Lynne Maxwell

USA

Colin Mccartney

Canada

William Mcgee

USA

Darryl Mcgill

Australia

Graeme Mcleod

United Kingdom

Vincent Minville

France

Sukanya Mitra

India

Jeffrey Mogil

Canada

Iain Moppett

United Kingdom

Hany Mowafi

Saudi Arabia

Eveline Nueesch

United Kingdom

Doris Østergaard

Denmark

Owen O'Sullivan

Ireland

Stephen Pastores

USA

Rupert Pearse

United Kingdom

Anders Perner

Denmark

Ville Pettilä

Finland

Philip Peyton

Australia

Hemanshu Prabhakar

India

Douglas Raines

USA

Ramachandran Ramani

USA

Saifudin Rashiq

Canada

Jochen Renner

Germany

Daniel Reuter

Germany

Emanuel Rivers

USA

Edgar Alfonso Romero-Sandoval

USA

Meg Rosenblatt

USA

Leif Saager

USA

Sanna Salanterä

Finland

Michael Sander

Germany

Volkher Scharnhorst

Netherlands

Stefan Schraag

United Kingdom

Frederique Servin

France

Rajeev Sharma

India

Tatjana Simurina

Croatia

Frank Skorpen

Norway

Peter Smitham

United Kingdom

Luzius A Steiner

Switzerland

Konstantinos Stroumpoulis

Greece

Bala Subramaniam

USA

Anna Taddio

Canada

Yoshihiro Takasugi

Japan

Mark Tommerdahl

USA

Angela Truong

USA

David Turnbull

United Kingdom

Todd Vanderah

USA

Luigi Vetrugno

Italy

Juan Viñoles

Spain

Thomas Volk

Germany

Christian Von Heymann

Germany

Deborah Wagner

USA

Zen'Ichiro Wajima

Japan

Thomas Weber

Austria

Manuel Wenk

Germany

Chris Winkelman

USA

Sandhya Yaddanapudi

India

Wei-Hsian Yin

Taiwan

## Pre-publication history

The pre-publication history for this paper can be accessed here:

http://www.biomedcentral.com/1471-2253/13/3/prepub

